# P-1852. Investigating the infant gut microbiome in relation to R21/Matrix-M™ vaccine immunogenicity and efficacy

**DOI:** 10.1093/ofid/ofae631.2013

**Published:** 2025-01-29

**Authors:** Mehreen S Datoo, Sandi Yen, Nick Edwards, Ousmane Traore, Athanase Some, Hamtandi M Natama, Jethro Johnson, Adrian V S Hill, Halidou Tinto

**Affiliations:** University of Oxford, Oxford, England, United Kingdom; University of Oxford, Oxford, England, United Kingdom; University of Oxford, Oxford, England, United Kingdom; Unité de Recherche Clinique de Nanoro, Institut de Recherche en Sciences de la Santé, Nanoro, Burkina Faso, Nanoro, Boulkiemde, Burkina Faso; Unité de Recherche Clinique de Nanoro, Institut de Recherche en Sciences de la Santé, Nanoro, Burkina Faso, Nanoro, Boulkiemde, Burkina Faso; Unité de Recherche Clinique de Nanoro, Institut de Recherche en Sciences de la Santé, Nanoro, Burkina Faso, Nanoro, Boulkiemde, Burkina Faso; University of Oxford, Oxford, England, United Kingdom; University of Oxford, Oxford, England, United Kingdom; Unité de Recherche Clinique de Nanoro, Institut de Recherche en Sciences de la Santé, Nanoro, Burkina Faso, Nanoro, Boulkiemde, Burkina Faso

## Abstract

**Background:**

R21/Matrix-M™ malaria vaccine was recently licensed in young children following good vaccine efficacy and induction of high titre antibody responses to the central repeat (NANP) of the P. *falciparum* circumsporozoite protein. Variability in NANP responses and efficacy was noted in the populations assessed and according to age.

Links between the gut microbiome and the immune system mean early life gut microbiome is an important consideration when understanding vaccine responses. We investigated the relationship of these and participant factors with the gut microbiome in Burkinabe infants in the R21/Matrix-M™ phase IIb trial.

**Methods:**

A phase IIb double-blind, randomised controlled trial assessing safety, immunogenicity and efficacy of R21/Matrix-M™ in 5-17 month olds took place in Nanoro, Burkina Faso from 2019 to 2023. Rectal swabs were collected at baseline and a year later, for both malaria vaccine (n=223) and control participants (n=77). Participant factors (i.e. age, gender, feeding, number of siblings, and Z-score), malaria episodes, and anti-NANP antibody responses were measured. Microbiome was assessed using 16S rRNA gene sequencing. Microbiome alpha- and beta- diversity were characterized, and microbial abundances were analysed using univariate and multivariate models.

**Results:**

Participant microbiomes showed two discernible compositional phenotypes at baseline and follow-up timepoints. The microbiome of majority of participants (93%) were predominantly *Firmicutes* (39%) and *Bacteroidetes* (37%) and low in *Proteobacteria* (13%). However, a subset of participants (7%) had microbiomes that were mainly *Proteobacteria* (63%). Within-individual alpha-diversity significantly varied with age, but not other participant factors. Relative abundances of certain bacteria were associated with age. Microbial abundances will be analysed and presented against antibody responses, as well as the number of clinical malaria episodes in the first two years.

**Conclusion:**

We describe the microbiome of Burkinabe infants within the context of R21/Matrix-M™ malaria vaccination. This dataset provides insight into a previously underrepresented population during early life, which is a key window for immunological and microbiome development.

**Disclosures:**

**Adrian V S Hill, PhD**, Serum Institute of India: Grant/Research Support|Serum Institute of India: I am named as a co-inventor on patent applications related to R21

